# *Paramicrobacterium salitolerans* sp. nov. isolated from the agricultural soil and *Microbacterium fluminis* sp. nov. isolated from the Han River, South Korea

**DOI:** 10.71150/jm.2512014

**Published:** 2026-03-05

**Authors:** Gracia Pradnya Lolita, Do-Hoon Lee, Yong-Seok Kim, Chang-Jun Cha

**Affiliations:** Department of Systems Biotechnology, Chung-Ang University, Anseong 17546, Republic of Korea

**Keywords:** *Paramicrobacterium*, *Microbacterium*, *Paramicrobacterium salitolerans*, *Microbacterium fluminis*, genome

## Abstract

Two novel bacterial species, designated as CJ85^T^ and CJ88^T^, were isolated from the agricultural soil and the Han River, South Korea, respectively. Cells of both strains were Gram-staining-positive, short rod-shaped, non-motile, and yellow-pigmented. Strain CJ85^T^ exhibited optimal growth in tryptic soy broth at 37°C and pH 7.0 in the absence of NaCl. Strain CJ88^T^ showed optimal growth in lysogeny broth at 30°C and pH 7.0 in the absence of NaCl. Phylogenetic analysis based on 16S rRNA gene sequences revealed that strain CJ85^T^ belonged to the genus *Paramicrobacterium*, showing the highest sequence similarity to *Paramicrobacterium fandaimingii* HY82^T^ (97.6%). Strain CJ88^T^ was assigned to the genus *Microbacterium*, with the highest sequence similarity to *Microbacterium azadirachtae* DSM 23848^T^ (98.5%). The DNA G + C content was 64.8% for strain CJ85^T^ and 70.5% for strain CJ88^T^. The genome-based analyses, including phylogenomic tree, digital DNA-DNA hybridization, and average nucleotide identity, clearly indicated that these strains represent novel species within their respective genera. The major fatty acids of both strains were *anteiso*-C_15:0_, *anteiso*-C_17:0_, and *iso*-C_16:0_. Based on the polyphasic taxonomy study, strains CJ85^T^ and CJ88^T^ represent novel species of the genera *Paramicrobacterium* and *Microbacterium*, respectively, for which names *Paramicrobacterium salitolerans* sp. nov. and *Microbacterium fluminis* sp. nov. are proposed. The type strains CJ85^T^ (= KACC 23064^T^ = JCM 36217^T^) and CJ88^T^ (= KACC 24080^T^ = JCM 38050^T^).

## Introduction

Plant-pathogenic bacteria belonging to the family *Microbacteriaceae* within the phylum *Actinomycetota* were first reported about a century ago (Smith, 1910, 1913) and the type species of the type genus of the family, *Microbacterium lacticum* isolated from dairy products was described afterwards (Orla-Jensen, 1919). Members of this family are Gram-positive, predominantly slender irregular rods, colored in colonies due to carotenoid pigments, chemo-organotrophic, aerobic, catalase-positive, mesophiles, and neutrophiles (Evtushenko, 2015). According to the List of Prokaryotic names with Standing in Nomenclature (LPSN), the family *Microbacteriaceae* currently encompasses 69 genera with validly published names (Parte et al., 2020).

The genus *Microbacterium* was proposed by Orla-Jensen (1919), of which the description was emended by Collins et al. (1983) and emended again by Takeuchi and Hatano (1998) to unite *Microbacterium* and *Aureobacterium*. The members of the genus *Microbacterium* have been isolated from diverse habitats such as seawater (Kim et al., 2008), soil (Lee et al., 2014), air (Zlamala et al., 2002), plants (Behrendt et al., 2001), and human blood (Clermont et al., 2009). They are characterized by the presence of *N*-glycolyl residues in the cell wall, menaquinone-11 (MK-11), MK-12, and MK-13 as primary respiratory quinones. The polar lipids consist of diphosphatidylglycerol, phosphatidylglycerol and one or more glycolipids; cellular fatty acids are predominantly *anteiso*-C_15:0_, *anteiso*-C_17:0_, and *iso*-C_16:0_ (Suzuki and Hamada, 2015). The genomic DNA G + C content ranged 61.6–71.8% (Lee et al., 2023). Currently, a total of 160 species with validly published names are reported under the genus *Microbacterium*, according to the LPSN (Parte et al., 2020).

The genus *Paramicrobacterium* was first proposed by Lee et al. (2023). The four members of genus *Microbacterium*–*M. agarici*, *M. chengjingii*, *M. fandaimingii*, and *M. humi*–were transferred to the genus *Paramicrobacterium* based on the phylogenomic analysis, including average nucleotide identity (ANI), average amino acid sequence identity (AAI), and digital DNA-DNA hybridization (dDDH) values (Lee et al., 2023). The members of the genus *Paramicrobacterium* are isolated from animals (Young et al., 2010) and plants (Zho et al., 2021). Chemotaxonomic characteristics of *Paramicrobacterium* were almost similar to those of *Microbacterium* (Lee et al., 2023). The genomic DNA G + C content values of *Paramicrobacterium* were 62.0–64.6% (Lee et al., 2023). During this study, we initially isolated two strains belonging to the genus *Microbacterium* but, strain CJ85^T^ was transferred to the newly proposed genus *Paramicrobacterium*. Based on the polyphasic taxonomy and genomic approaches, strains CJ85^T^ and CJ88^T^ are proposed as novel species of the genus *Paramicrobacterium* and *Microbacterium* which isolated from the lettuce cultivation soil in Yongin-si and the Han River, South Korea.

## Materials and Methods

### Isolation of bacterial strains and culture conditions

Strains CJ85^T^ and CJ88^T^ were isolated from a lettuce cultivation soil in Yongin (37°9'30.67"N, 127°17'20.29"E) and from a freshwater sample of the Han River (37°24'5.45"N, 127°32'21.77"E), South Korea, respectively. Surface river water (0–1 m depth) and lettuce cultivation soil (5–10 cm depth) were collected. Each sample was inoculated into Reasoner’s 2A (R2A) broth (BD) at 30°C with shaking at 200 rpm. The resulting cultures were serially diluted and spread onto R2A agar. Single colonies were subsequently picked and purified through successive streaking on R2A agar at 30°C. For long-term storage, strains CJ85^T^ and CJ88^T^ were grown in R2A broth at 30°C for two days and subsequently maintained at –80°C in 30% (w/v) glycerol suspension.

### Phylogeny analysis based on 16S rRNA genes

A single colony of each strain served as the template for PCR amplification of the 16S rRNA gene. The amplicons were sequenced at Biofact (Daejeon, Korea) using an automated DNA analyzer and sequencing primers 27F, 1492R, 785F, and 805R (Baker et al., 2003). The sequences were assembled to generate a consensus sequence for each strain. Alignment between the consensus sequence of each strain and those of related type strains selected from Ezbiocloud database (https://www.ezbiocloud.net/) (Chalita et al., 2024) was executed through the MUSCLE program (Edgar, 2004). Phylogenetic trees were constructed using the neighbor-joining (NJ), maximum-parsimony (MP), and maximum-likelihood (ML) methods. The NJ and MP trees were constructed using MEGA 12 software (Kumar et al., 2024). For NJ trees, evolutionary distances were calculated using the Jukes-Cantor model (Jukes and Cantor, 1969). The MP trees were inferred using the pairwise deletion option for gaps. The ML trees were reconstructed using IQ-TREE, and the best-fit substitution model was determined by ModelFinder Plus under the Bayesian Information Criterion (Minh et al., 2020). The selected model for strain CJ85^T^ was the Transitional Model 3 with empirical base frequencies (+F) and a discrete Gamma distribution with three categories (+R3). For strain CJ88^T^, the model was the Transitional Model with empirical base frequencies (+F), a proportion of invariable sites (+I), and a discrete Gamma distribution with four rate categories (+G4). The topologies of all trees were evaluated based on bootstrap analysis of 1,000 datasets (Felsenstein, 1985).

### Whole genome sequencing and de novo assembly

The extraction of genomic DNA from each strain was carried out using DNeasy Blood & Tissue Kits (QIAGEN, Germany) in accordance with the manufacturer’s protocols. Whole genome sequencing was performed at JS LINK (Korea) using a hybrid approach combining Oxford Nanopore and Illumina platforms. For long-read sequencing, a genomic DNA library was prepared using the Ligation Sequencing Kit (SQK-LSK109; Oxford Nanopore, UK) and sequenced on an Oxford Nanopore MinION instrument with R9.4.1 flow cells (FLO-MIN106; Oxford Nanopore). For short-read sequencing, a separate library preparation was performed using the TruSeq DNA PCR-Free Kit and sequenced on an Illumina NovaSeq 6000 platform. The resulting Nanopore and Illumina reads were subject to de novo hybrid genome assembly with Unicycler v0.5.0 (Wick et al., 2017). The completeness of the final genome assemblies was assessed using BUSCO v5.3.2 against the *Microbacteriaceae* single-copy ortholog dataset (Manni et al., 2021).

### Phylogenomic analysis and genome-based characterization

To comply with the minimal standards for prokaryotic genomic taxonomy recommended by Chun et al. (2018), we estimated dDDH values through the Genome-to-Genome Distance Calculator (GGDC 3.0) (Meier-Kolthoff et al., 2022). ANI values were calculated using the OrthoANI pipeline (Lee et al., 2016). For genus-level classification, AAI values were calculated with the EzAAI software (Kim et al., 2021b), and the percentage of conserved proteins (POCP) values were determined as described by Qin et al. (2014). Reference genome sequences of closely related type strains were obtained from the national center for biotechnology information (NCBI) type material database (O'Leary et al., 2024). The up-to-date bacterial core genes 2 (UBCG2) pipeline was used to generate the concatenated alignment of 81 UBCG sequences (Kim et al., 2021a). Phylogenomic trees were constructed from the concatenated sequences using IQ-TREE with the general time reversible substitution model, empirical base frequency (+F), and the FreeRate model with ten categories (+R10) (Minh et al., 2020). Phylogenemic tree was visualized using iTOL v7 (Letunic and Bork, 2024). Protein-coding sequences (CDSs), rRNA genes, and tRNA genes were predicted and functionally annotated using Bakta (Schwengers et al., 2021). Functional metabolic pathways, carbohydrate-active enzymes, and proteolytic enzymes were predicted using the KEGG, CAZy, MEROPS databases, respectively, implemented in METABOLIC software (Zhou et al., 2022). Pan- and core-genome analyses were conducted using Panaroo pipeline (Tonkin-Hill et al., 2020). Antimicrobial resistance genes (ARGs) were identified based on the Comprehensive Antibiotic Resistance Database using the Resistance Gene Identifier with strict cutoff (Alcock et al., 2023).

### Morphological and physiological characterization

Cell morphology was observed using transmission electron microscopy with cells grown on tryptic soy agar (TSA) at 30°C for 24 h. Growth was assessed in R2A broth, tryptic soy broth (TSB), Luria-Bertani (LB) broth, nutrient broth (NB), and marine broth (MB). The optimal growth temperature was determined on TSA (for strain CJ85^T^) and LB agar (for strain CJ88^T^) at temperatures ranging from 4 to 50°C (4, 10, 20, 25, 30, 37, 45, and 50°C). The pH range for growth was tested in TSB and LB broth media buffered to pH 4.0–5.0 (0.1 M citrate), pH 6.0–8.0 (0.1 M phosphate), and pH 9.0–10.0 (0.1 M bicarbonate-carbonate). NaCl tolerance was assessed in TSB and LB broth supplemented with 0.0–15% (w/v) NaCl in 1.0% increments. The pH and NaCl tolerance tests were carried out at 30°C for up to two days. Gram-staining was carried out using a commercial kit from Sigma-Aldrich, following the manufacturer’s protocols. Cell motility was examined by stab-inoculating a semi-solid medium (TSB or LB broth with 0.4% agar) and observing growth after seven days of incubation. Anaerobic growth was evaluated by incubating strains CJ85^T^ and CJ88^T^ on TSA and LB agar, respectively, at their optimal temperature for two weeks using the GasPak Anaerobe Pouch System (BD). Oxidase and catalase activities were determined using oxidase reagent (bioMérieux) and 3.0% (v/v) H_2_O_2_ solution, respectively. Hydrolysis of starch (1.0% w/v), cellulose (0.2% w/v, carboxymethyl cellulose), casein (3.0% w/v, skim milk), and DNA (DNase test agar, BD) was assessed after seven days of incubation. Carbon source utilization and additional enzymatic activities were determined using API 20NE kit according to the manufacturer’s instructions.

### Chemotaxonomic analyses

For chemotaxonomic analyses, cells of strain CJ85^T^ and CJ88^T^ were cultured on TSA and LB agar, respectively, for 24 h at their optimal temperatures. The polar lipids were extracted from 100 mg of freeze-dried cells and analyzed by two-dimensional silica gel thin-layer chromatography (TLC) as described by Minnikin et al. (1984). For the first-dimension separation, a solvent mixture of chloroform:methanol:water (32.5:12.5:2, v/v/v) was employed, followed by a second dimension developed with chloroform:methanol:acetic acid:water (40:6:7.5:2, v/v/v/v). Separated polar lipids were visualized by spraying with specific reagents: 5% (w/v) phosphomolybdic acid for total lipids, molybdenum blue spray reagent (Sigma-Aldrich) for phospholipids, 0.25% (w/v) ninhydrin for aminolipids, and 15% (w/v) α-naphthol for glycolipids. For peptidoglycan analysis, whole cells were hydrolyzed in 6 N HCl at 121°C for 15 min, and the hydrolysates were analyzed by TLC on silica plates using the solvent system of Hasegawa et al. (1983). Respiratory quinones were extracted as described by Minnikin et al. (1984) and analyzed by high-performance liquid chromatography on a reverse phase column (Apollo C18, 250 × 4.6 mm, 5 μm) (Collins, 1985). Quinones were eluted with a methanol:iso-propanol mixture (4:1, v/v) at a flow rate of 1 ml/min and detected by UV absorbance at 270 nm. Cellular fatty acids were saponified, methylated, extracted, and analyzed by gas chromatography (Hewlett Packard 6890) using the standard protocol of the Microbial Identification System (Sherlock ver. 6.0B; RTSBA6 database) (Sasser, 1990).

### Antimicrobial susceptibility test

The antimicrobial susceptibility was evaluated by the disk diffusion assay using antibiotic impregnated disks (Lioflchem, Italy). The following antibiotics were tested (disk content in μg): chloramphenicol (30), ciprofloxacin (5), clindamycin (2), erythromycin (15), fosfomycin (200), gentamicin (10), linezolid (10), rifampicin (5), streptomycin (10), tetracycline (30), trimethoprim (5), and vancomycin (30). Results were interpreted as susceptible (S), intermediate (I), or resistant (R) according to the clinical breakpoints for *Staphylococcus* spp. and *Enterococcus* spp. provided in the CLSI breakpoint table (Lewis II et al., 2025).

### GenBank accession numbers

The GenBank accession numbers for the 16S rRNA gene sequences of strains CJ85^T^ and CJ88^T^ are PV837006 and PV837007, respectively, and for the genome sequences are GCA_051513965.1 and GCA_051549805.1, respectively.

## Results and Discussion

### 16S rRNA phylogeny

**Fig. 1.**
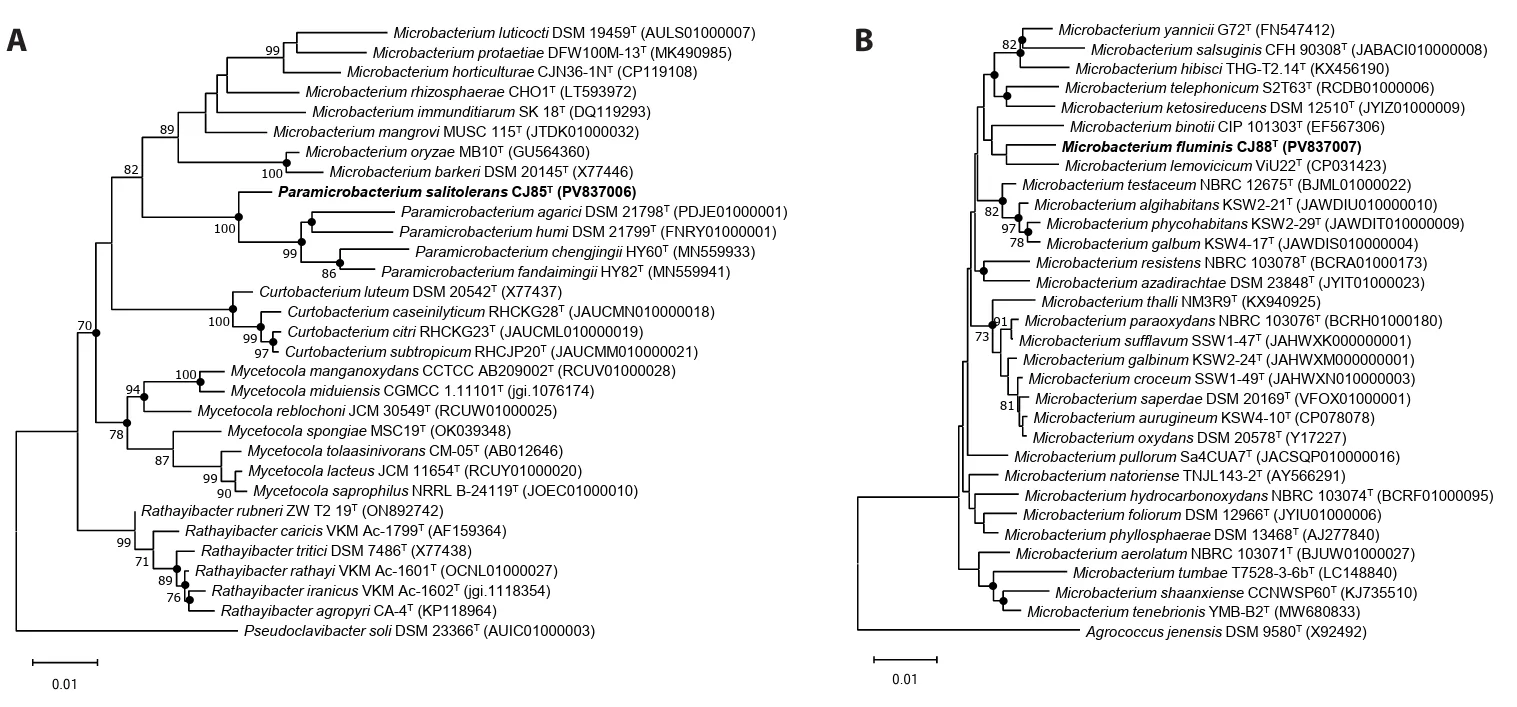
Neighbor-joining phylogenetic trees of strains CJ85^T^ (A) and CJ88^T^ (B) and their related type strains based on 16S rRNA gene sequences. Closed circles indicate nodes recovered in the three trees generated by neighbor-joining, maximum-likelihood, and maximum-parsimony methods. Bootstrap values greater than 70% are shown at branch points based on 1,000 replicated datasets. *Pseudoclavibacter soil* DSM 23366^T^ (A) and *Agrococcus jenensis* DSM 9580^T^ (B) were used as an outgroup, respectively. Bars, 0.01 substitutions per nucleotide position.

Phylogenetic analysis based on the 16S rRNA gene sequences revealed that strain CJ85^T^ formed a robust clade with members of the genus *Paramicrobacterium*, displaying the highest sequence similarity with *P. fandaimingii* HY82^T^ (97.7%), followed by *P. agarici* DSM 21798^T^ (97.5%) and *P. chengjingii* HY60^T^ (97.4%; Fig. 1A); the topologies of the NJ, ML, and MP trees were congruent (Figs. 1A and S1). Strain CJ88^T^ clustered with *M. lemovicicum* DSM 25044^T^ with the highest 16S rRNA gene sequence similarity of 98.4% (Fig. 1B); however, the phylogenetic position of strain CJ88^T^ was not consistent across the different analytical methods, showing a different topology in the MP tree compared to the NJ and ML trees (Figs. 1B and S2). Based on pairwise 16S rRNA gene sequence identities below the established threshold for species delineation (Kim et al., 2014; Rosselló-Móra and Amann, 2015), strains CJ85^T^ and CJ88^T^ represent novel species within the genus *Paramicrobacterium* and *Microbacterium*, respectively.

### Genomic features

**Fig. 2.**
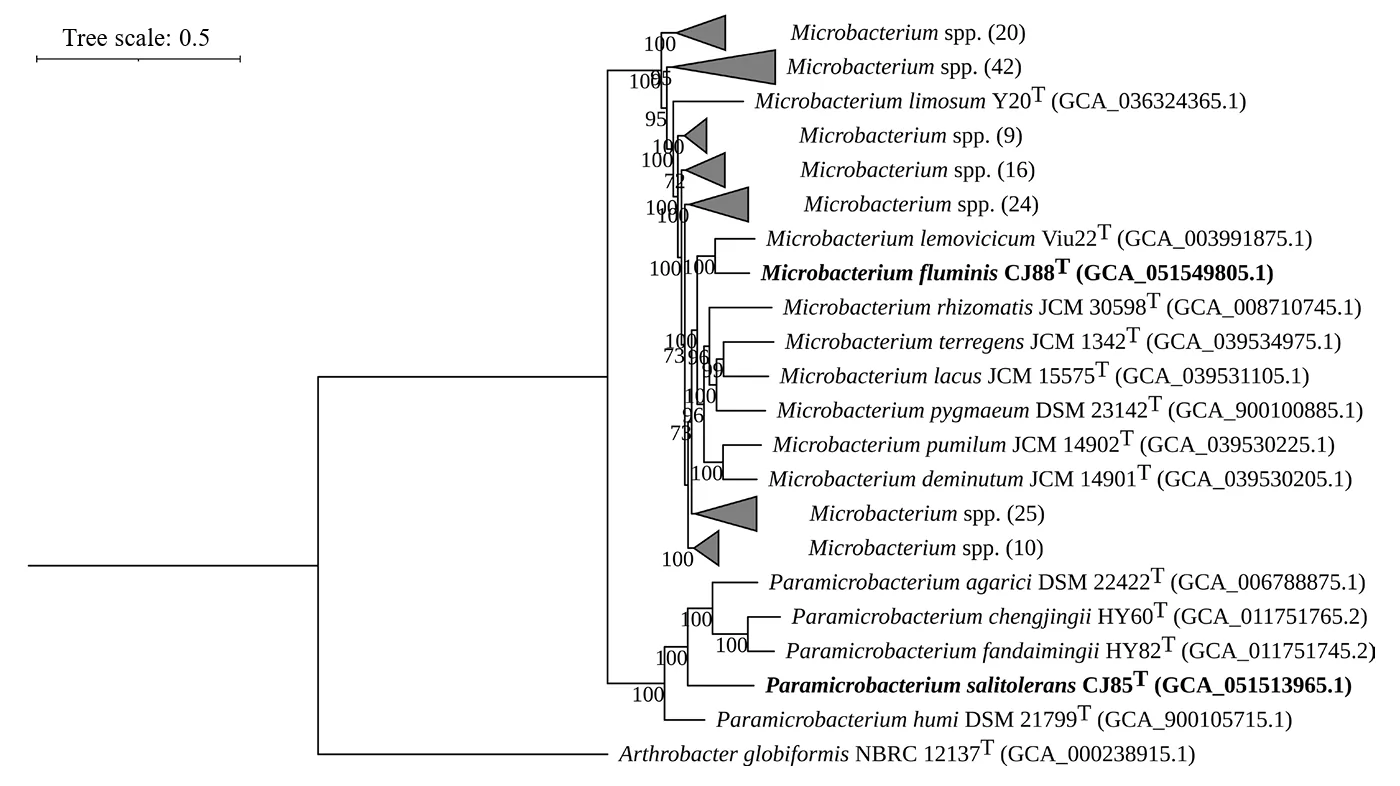
Phylogenomic tree of strains CJ85^T^ and CJ88^T^ and their related type strains based on the concatenated sequences of 81 UBCGs. This is a summarized phylogenomic tree of the whole phylogenomic tree reconstructed using 158 type strains belonging to the genera *Paramicrobacterium* and *Microbacterium*. The whole phylogenomic tree is provided in Fig. S4. Numbers in parentheses indicate the number of species included in each collapsed clade. Bootstrap values greater than 70% are shown at branch points based on maximum-likelihood analysis of 1,000 replicated datasets. The *Arthrobacter globiformis* NBRC 12137^T^ was used as an outgroup. Bar, 0.5 substitutions per nucleotide position.

The genome assemblies of strains CJ85ᵀ and CJ88ᵀ exhibited 98.7% and 99.5% completeness (Fig. S3), with genome sizes of 3.17 Mbp and 3.24 Mbp, respectively. The genomic DNA G + C contents were 64.8% for strain CJ85^T^ and 70.5% for strain CJ88^T^. The genome of strain CJ85^T^ was predicted to contain 3,012 CDSs, 47 tRNA genes, and 6 rRNA genes, while that of strain CJ88^T^ contained 3,065 CDSs, 48 tRNA genes, and 6 rRNA genes. Both strains harbored two identical copies of the 16S rRNA genes. The genomic characteristics of both strains and their related type strains are summarized in Table 1. A phylogenomic tree constructed using the concatenated sequences of 81 UBCGs from the two novel strains and 158 related type strains (*Microbacterium* and *Paramicrobacterium*) showed that strain CJ85^T^ clustered within the genus *Paramicrobacterium*, forming a robust monophyletic clade with *P. fandaimingii* HY82^T^, *P. agarici* DSM 21798^T^, and *P. chengjingii* HY60^T^. Strain CJ88^T^ was positioned within the genus *Microbacterium*, and indicated the closest phylogenetic relationship to *M. lemovicicum* DSM 25044^T^, complementing the incongruence of 16S rRNA gene-based phylogeny (Figs. 2 and S4).

**Fig. 3.**
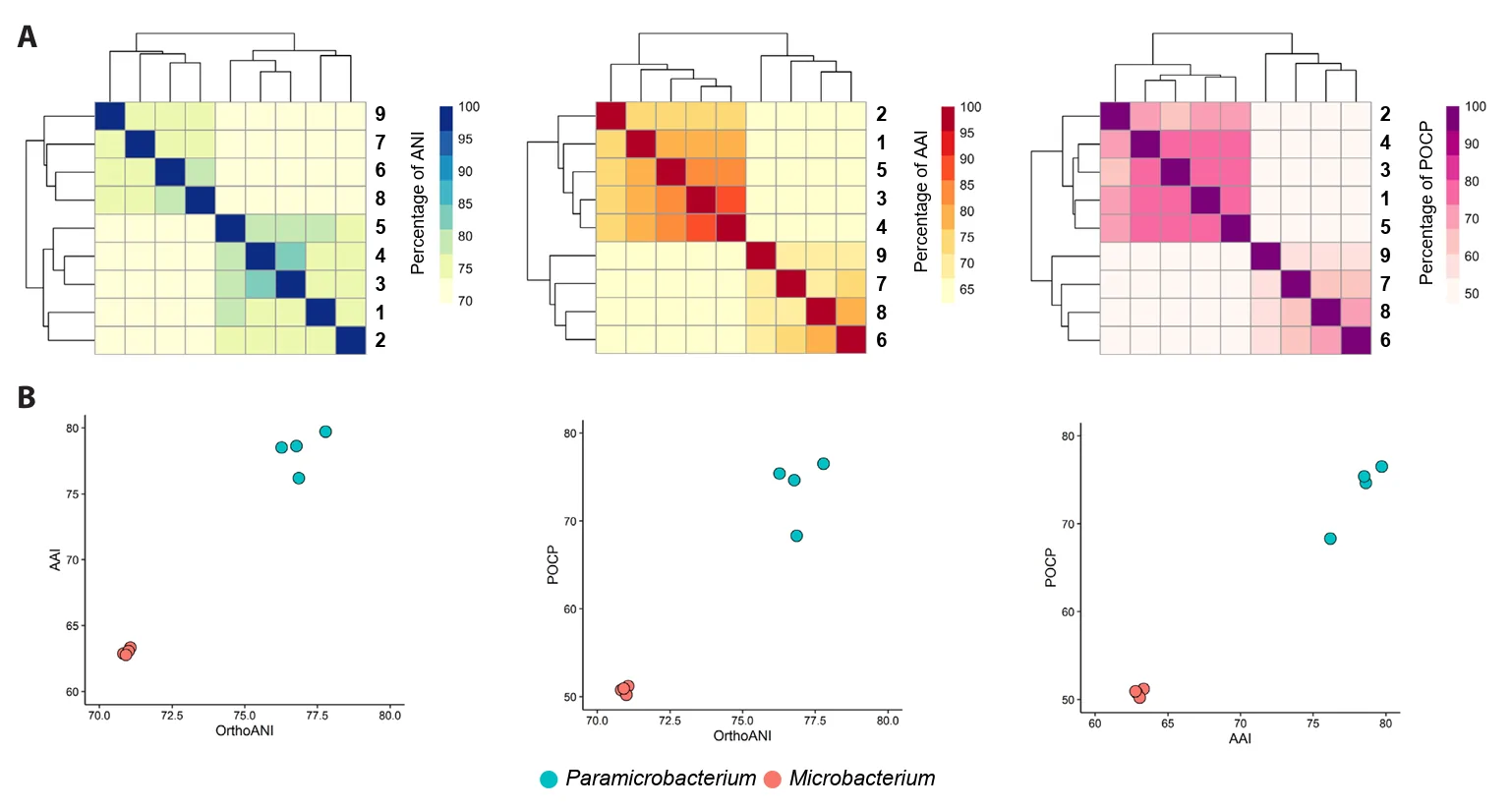
Overall genomic relatedness indices (OGRIs) of strains CJ85^T^ and CJ88^T^ and related type strains. (A) Heatmaps showing pairwise values for OrthoANI (blue), AAI (red), and POCP (purple) values among genomes of the genera *Paramicrobacterium* and *Microbacterium*. The dendrograms indicate hierarchical clustering of the genomes based on each OGRI. (B) Correlation plots showing the OGRI values calculated between *Paramicrobacterium* and *Microbacterium* species. Each value represents the set of values for a pairwise comparison against CJ85^T^, with the 100% self-comparison value excluded. Strains: 1, *P. salitolerans* CJ85^T^; 2, *P. humi* DSM 21799^T^; 3, *P. chengjingii* HY60^T^; 4, *P. fandaimingii* HY82^T^; 5, *P. agarici* DSM 21798^T^; 6, *M. fluminis* CJ88^T^; 7, *M. binotii* JCM 16365^T^; 8, *M. lemovicicum* ViU22^T^; 9, *M. azadirachtae* DSM 23848^T^.

For taxonomic classification, a suite of genomic indices was calculated. The ANI values between strain CJ85^T^ and its closest related type strains, *P. fandaimingii* HY82^T^, *P. agarici* DSM 21798^T^, *P. chengjingii* HY60^T^, and *P. humi* DSM 21799^T^, were 76.8%, 77.8%, 76.3%, and 76.9%, respectively (Table S1). The ANI values between strain CJ88^T^ and *M. azadirachtae* DSM 23848^T^, *M. lemovicicum* ViU22^T^, and *M. binotii* JCM 16365^T^ were 75.9%, 80.4%, and 76.9%, respectively (Table S1). All dDDH values between the novel strains and their related type strains were below 30% (Table S1). These results indicate that both strains represent a novel species within the genera *Paramicrobacterium* and *Microbacterium*, respectively, based on genomic ANI (< 95–96%) and dDDH values (< 70%) (Chun et al., 2018; Meier-Kolthoff et al., 2013; Richter and Rosselló-Móra, 2009). Furthermore, AAI and POCP values revealed a clear distinction between strains CJ85^T^ and CJ88^T^ and their related type strains at the genus level (Fig. 3, Table S2), supporting the separation of *Paramicrobacterium* from *Microbacterium*. Pangenome analysis of species of *Paramicrobacterium* and *Microbacterium* identified 1,711 and 963 core orthologous genes, respectively, whereas a combined analysis of both genera identified a shared core genome of 540 orthologous genes (Fig. S5).

Two glycopeptide ARGs (*vanYB* and *vanYA*) were predicted in the genome of strain CJ88^T^. However, the predicted resistance genotype was not consistent with the observed phenotype (Table S3). KEGG pathway analysis revealed that the genome of strains CJ85^T^ and CJ88^T^ encode 65 and 58 complete metabolic pathway modules, respectively (Fig. S6, Table S4). Both strains possess well-conserved central carbohydrate metabolism pathways, including glycolysis, glucogenesis, pentose phosphate pathway, phosphoribosyl diphosphate biosynthesis, and citrate cycle (Fig. S6). Notably, pathways for ectoine biosynthesis and β-lactam resistance (*bla* system) were found exclusively in genus *Paramicrobacterium*. Analysis of carbohydrate-active enzymes showed that the genome of strain CJ85^T^ encoded two polysaccharide lyases (PLs) and 45 glycoside hydrolases (GHs), whereas the strain CJ88^T^ genome encoded 22 GHs with no PL (Fig. S7A). Specifically, the genome of strain CJ85^T^ harbored unique GH18 and GH19 families (Fig. S7B), which were absent from other related type strains. Strain CJ88^T^ exhibited no distinct GH family profile compared to other related type strains (Fig. S7B). Both strains and the other related type strains all possessed GH enzymes required for cellulose degradation, suggesting that GH family profile is not consistent with the phenotypic characteristic of cellulose degradation (Fig. S7, Table 2); functional cellulolytic activity likely results from the synergistic action of multiple GH enzymes (Contato et al., 2025). The higher number of GH genes present in the CJ85^T^ genome suggests that strain CJ85^T^ possesses genomic content more optimized for growth in soil habitats compared to the freshwater-derived strain CJ88^T^. Analysis of proteolytic enzymes revealed that metallo, serine, and cysteine proteases were the predominant proteolytic enzymes in both strains (Fig. S8). Regarding flagellar biosynthesis genes, *flhA* gene was identified in the genome of strain CJ85^T^, whereas no flagellar-related gene was found in strain CJ88^T^. However, since the assembly of a functional flagellum requires a complex apparatus consisting of numerous proteins (Worrall et al., 2023), the presence of *flhA* gene alone is insufficient for flagellar formation. These genomic findings are consistent with the non-flagellated morphology of both strains, as observed in transmission electron microscopy images (Fig. S9).

### Characterization of morphological, phenotypic, and biochemical features

Strains CJ85^T^ and CJ88^T^ were Gram-stain-positive, strictly aerobic, non-motile, and non-spore-forming rods. After 24 h of cultivation, colonies of strain CJ85^T^ on TSA at 37°C were smooth, light-yellow, and circular, while colonies of CJ88^T^ on LB agar at 30°C were smooth, yellow-pigmented, and circular. Transmission electron micrographs showed that both cells were rod-shaped (Fig. S9). Optimal growth for CJ85^T^ was observed on TSA and for strain CJ88^T^ on LB agar; no growth was observed for either strain on marine broth. Strain CJ85^T^ grew at 10–45°C (optimum 37°C) and at pH 5.0–9.0 in the presence of 0.0–14.0% (w/v) NaCl. Strain CJ88^T^ grew at 10–45°C (optimum 30°C) and at pH 6.0–9.0 in the presence of 0.0–3.0% (w/v) NaCl. For both strains, optimal growth was perceived at pH 7.0 in the absence of NaCl. Differential biochemical and physiological characteristics between the novel strains and their closely related type strains are provided in Table 2.

### Chemotaxonomic characteristics

The predominant respiratory quinone was identified as MK-11 for strain CJ85^T^ and MK-12 for strain CJ88^T^. The cell-wall peptidoglycan of both strains contained ornithine and meso-diaminopimelic acid. As shown in Table 3, the major cellular fatty acids of strain CJ85^T^ were *anteiso*-C_15:0_ (42.3%), *iso*-C_16:0_ (22.7%), and *anteiso*-C_17:0_ (22.1%; Table 3). The polar lipids of strain CJ85^T^ included diphospatidylglycerol, phosphatidylglycerol, two glycolipids, and two unidentified polar lipids (Fig. S10A). For strain CJ88^T^, the major cellular fatty acids were *anteiso*-C_15:0_ (40.2%), *iso*-C_16:0_ (21.5%), and *anteiso*-C_17:0_ (19.2%; Table 3). The polar lipids of strain CJ88^T^ included diphospatidylglycerol, phosphatidylglycerol, glycolipid, and one unidentified polar lipid. Phosphatidylethanolamine was not detected in either strain (Fig. S10B). These chemotaxonomic features are consistent with those of their related genera within the family *Microbacteriaceae*.

### Taxonomic conclusion

Genomic, phylogenetic, chemotaxonomic, and phenotypic analyses demonstrated that strain CJ85^T^ belongs to the genus *Paramicrobacterium* and strain CJ88^T^ belongs to the genus *Microbacterium*. The observed characteristics of both strains, including respiratory quinones, fatty acids, polar lipids, and carbon source utilization patterns, were consistent with the descriptions of their related species of each genus. For strain CJ85^T^, several characteristics such as positive oxidase and urease activity, hydrolysis of cellulose, and the absence of the dTDP-L-rhamnose biosynthesis module, distinguished the novel strain from its related type strains. Strain CJ88^T^ was differentiated by its negative β-galactosidase activity and hydrolysis of DNA, whereas its related type strains were positive. In contrast, strain CJ88^T^ exhibited positive hydrolysis of cellulose and casein, whereas its related type strains were negative. Based on genomic analyses, the low dDDH (< 70%) and ANI (< 95–96%) values clearly differentiated strains CJ85^T^ and CJ88^T^ from all known species of each genus. Consequently, based on the polyphasic taxonomy analyses, strain CJ85^T^ represents a novel species of the genus *Paramicrobacterium*, for which the name *Paramicrobacterium salitolerans* sp. nov. is proposed and strain CJ88^T^ represents a novel species of the genus *Microbacterium*, for which the name *Microbacterium fluminis* is proposed.

### Description of *Paramicrobacterium salitolerans* sp. nov.

*Paramicrobacterium salitolerans* (sa.li.to'le.rans. L. masc. n. *sal*, salt; L. pres. part. *tolerans*, tolerating; N.L. part. adj. *salitolerans*, salt-tolerating).

Cells are aerobic, Gram-stain-positive, non-motile and rod-shaped. Colonies grown for 24 h on TSA at 37°C are light-yellow, convex, and smooth. Growth is observed at 10–45°C (optimum 37°C), at pH 5.0–9.0 (optimum pH 7.0) and in the presence of 0.0–14.0% NaCl (optimum 0.0%). Catalase- and oxidase-positive. Starch, carboxymethyl cellulose, DNA, and aesculin ferric citrate are hydrolyzed but casein and gelatin are not. Indole is not produced. Enzyme activities for urease and β-galactosidase are positive but negative for arginine dihydrolase. According to the API 20NE test, D-glucose, L-arabinose, D-mannose, D-mannitol, *N*-acetyl-glucosamine, D-maltose, and potassium gluconate are assimilated, but capric acid, adipic acid, malic acid, trisodium citrate, and phenylacetic acid are not. The predominant polar lipids are diphosphatidylglycerol, phosphatidylglycerol, two glycolipids, and two unidentified polar lipids. The major fatty acids are *anteiso*-C_15:0_, *iso*-C_16:0_, and *anteiso*-C_17:0_. The predominant respiratory quinone is MK-11. The type strain is CJ85^T^ (= KACC 23064^T^ = JCM 36217^T^), isolated from a lettuce cultivation soil in Yongin, Korea. The DNA G + C content of the type strain is 64.8%. The GenBank accession numbers for the 16S rRNA gene sequence and the whole genome sequence of strain CJ85^T^ are PV837006 and GCA_051513965.1, respectively.

### Description of *Microbacterium fluminis* sp. nov.

*Microbacterium fluminis* (flu'mi.nis. L. neut. n. *flumen*, a river; N.L. gen. n. *fluminis*, of a river).

Cells are aerobic, Gram-stain-positive, non-spore-forming, non-motile, and rod-shaped. Colonies grown for 24 h on LB agar at 30°C are yellow, convex, and smooth. Growth is observed at 10–45°C (optimum 30°C), at pH 6.0–9.0 (optimum pH 7.0) and in the presence of 0.0–3.0% NaCl (optimum 0.0%). Catalase- and oxidase-positive. Starch, carboxymethyl cellulose, casein, and aesculin ferric citrate are hydrolyzed but DNA and gelatin are not. Indole is not produced. Enzyme activity is negative for urease, β-galactosidase and arginine dihydrolase. According to the API 20NE test, D-glucose, D-mannose, D-mannitol, D-maltose, potassium gluconate, and malic acid are assimilated, but L-arabinose, *N*-acetyl-glucosamine, capric acid, adipic acid, trisodium citrate, and phenylacetic acid are not. The predominant polar lipids are diphospatidylglycerol, phosphatidylglycerol, glycolipid, and one unidentified polar lipid. The major fatty acids are *anteiso*-C_15:0_, *iso*-C_16:0_, and *anteiso*-C_17:0_. The predominant respiratory quinone is MK-12. The type strain is CJ88^T^ (= KACC 24080^T^ = JCM 38050^T^), isolated from the Han River, Korea. The DNA G + C content of the type strain is 70.5%. The GenBank accession numbers for the 16S rRNA gene sequence and the whole genome sequence of strain CJ88^T^ are PV837007 and GCA_051549805.1, respectively.

## Figures and Tables

**Table 1.** Genomic features of strains CJ85^T^ and CJ88^T^ with their related type strains of the genera *Paramicrobacterium* and *Microbacterium*. All genomic features were predicted and annotated using Bakta.

Strain	Accession number	Size (Mbp)	G + C contents (%)	Number of			
				contigs	protein coding genes	rRNAs	tRNAs
*P. salitolerans* CJ85^T^	GCA_051513965.1^†^	3.17	64.8	1	3,012	6	47
*P. fandaimingii* HY82^T^	GCA_011751745.2^‡^	3.62	63.3	1	3,464	6	47
*P. agarici* DSM 21798^T^	GCA_006788875.1^‡^	3.35	64.3	1	3,252	6	48
*P. chengjingii* HY60^T^	GCA_011751765.2^‡^	3.62	61.9	1	3,466	6	46
*P. humi* DSM 21799^T^	GCA_900105715.1^‡^	3.36	67.3	2	3,278	6	46
*M. fluminis* CJ88^T^	GCA_051549805.1^†^	3.24	70.5	1	3,065	6	48
*M. azadirachtae* DSM 23848^T^	GCA_000956545.1^‡^	4.04	70.5	86	3,755	5	45
*M. lemovicicum* ViU22^T^	GCA_003991875.1^‡^	3.56	70.9	1	3,297	6	48
*M. binotii* JCM 16365^T^	GCA_039532115.1^‡^	3.22	69.3	38	3,134	5	52

† Genome sequencing was conducted in this study.

‡ Genome sequences were retrieved from the National Center for Biotechnology Information.

**Table 2.** Differential characteristics of *Paramicrobacterium* strain CJ85^T^ and *Microbacterium* strain CJ88^T^ and their related type strains Strains: 1, *P. salitolerans* CJ85^T^; 2, *P. fandaimingii* HY82^T^; 3, *P. agarici* DSM 21798^T^; 4, *P. chengjingii* HY60^T^; 5, *P. humi* DSM 21799^T^; 6, *M. fluminis* CJ88^T^; 7, *M. azadirachtae* DSM 23848^T^; 8, *M. lemovicicum* ViU22^T^; 9, *M. binotii* JCM 16365^T^. All data are from this study unless indicated. All strains are positive for activity of catalase and aesculin hydrolysis. All strains are negative for indole production, activity of arginine dihydrolase, and assimilation of capric acid.

Characteristic	1	2	3	4	5	6	7	8	9
**Isolation source**	Freshwater	Bat feces	Mushroom	Bat feces	Mushroom	Soil	Rhizoplane	Soil	Human blood
**Colony color**	Light-yellow	White	Yellow	White	Yellow	Yellow	Light-yellow	Orange	Yellow
**Growth** ^*^									
Temperature (˚C)	10–45	10–30	20–37	10–30	20–37	10–45	10–37	15–37	15–37
pH	5–9	6–10	6–11	6–10	5–11	6–9	5–10	ND	ND
NaCl (%, w/v)	0.0–14.0	0.5–10.5	0.5–10.5	0.5–10.5	0.5–10.5	0.0–3.0	0.0–5.5	0.0–4.5	0.0–6.0
**Nitrate reduction**	+	+	+	+	+	−	+	−	−
**Fermentation of**									
Glucose	−	−	−	−	−	−	−	−	+
**Enzyme activity**									
Oxidase	+	−	−	−	−	+	−	+	−
Urease	+	−	−	−	−	−	−	−	−
β-Galactosidase	+	+	+	+	+	−	+	+	+
**Hydrolysis of**									
Starch	+	+	+	−	+	+	−	+	−
Cellulose	+	−	−	−	−	+	−	−	−
Casein	−	−	−	−	−	+	−	−	−
DNA	+	+	+	+	+	−	+	+	+
Gelatin	−	−	−	−	−	−	−	+	−
**Assimilation of**									
D-Glucose	+	+	+	−	+	+	+	+	+
L-Arabinose	+	−	+	−	+	−	+	−	+
D-Mannose	+	−	+	−	+	+	+	−	+
D-Mannitol	+	+	+	−	+	+	+	−	+
*N*-Acetyl-glucosamine	+	−	+	−	+	−	+	−	−
D-Maltose	+	−	+	−	+	+	+	+	+
Potassium gluconate	+	−	+	−	+	+	+	−	+
Adipic acid	−	−	+	−	+	−	−	−	+
Malic acid	−	−	+	−	+	+	+	−	+
Trisodium citrate	−	−	+	−	+	−	+	−	+
Phenylacetic acid	−	−	−	−	−	−	−	−	+

* Growth data are taken from the following sources: strains 2 and 4, Zho et al. (2021); strains 3 and 5, Young et al. (2010); strain 7, Madhaiyan et al. (2010); strain 8, Mondani et al. (2013); strain 9, Clermont et al. (2009).

Symbols: +, Positive; −, negative; ND, data not available.

**Table 3.** Fatty acid composition (%) of *Paramicrobacterium* strain CJ85^T^ and *Microbacterium* strain CJ88^T^ and the related type strains Strains: 1, *P. salitolerans* CJ85^T^; 2, *P. fandaimingii* HY82^T^; 3, *P. agarici* DSM 21798^T^; 4, *P. chengjingii* HY60^T^; 5, *P. humi* DSM 21799^T^; 6, *M. fluminis* CJ88^T^; 7, *M. azadirachtae* DSM 23848^T^; 8, *M. lemovicicum* ViU22^T^; 9, *M. binotii* JCM 16365^T^. All data are obtained from this study.

Fatty acid	1	2	3	4	5	6	7	8	9
**Straight**									
C_14:0_	TR	-	TR	-	TR	-	-	-	-
C_16:0_	2.5	-	3.9	-	1.6	TR	-	-	2.5
**Unsaturated**									
C_15:1_ *ω*5*c*	3.4	-	6.7	-	TR	-	-	-	-
**Branched**									
*iso*-C_10:0_	-	9.0	-	10.0	-	-	7.5	10.9	-
*iso*-C_14:0_	1.6	-	1.8	-	TR	TR	-	-	-
*iso*-C_15:0_	4.4	10.8	4.7	9.5	7.1	11.2	10.1	-	-
*iso*-C_16:0_	22.7	15.0	18.3	14.1	19.8	21.5	16.2	17.5	19.5
*iso*-C_17:0_	TR	-	1.3	-	2.6	5.0	3.6	-	-
*anteiso*-C_15:0_	42.3	44.3	43.5	39.7	41.0	40.2	34.9	50.5	39.7
*anteiso*-C_17:0_	22.1	17.7	17.7	17.5	23.4	19.2	23.8	13.8	35.8
*anteiso*-C_15:1_ A	-	-	1.3	-	-	-	-	-	-

Symbols: -, not detected; TR, trace amount (< 1.0%).

## Supplementary Information

Table S1. ANI and dDDH values between two novel strains and their related type strains. ANI values were shaded in blue and dDDG values in green color. Strains: 1, *P. salitolerans* CJ85^T^; 2, *P. fandaimingii* HY82^T^; 3, *P. agarici* DSM 21798^T^; 4, *P. chengjingii* HY60^T^; 5, *P. humi* DSM 21799^T^; 6, *M. fluminis* CJ88^T^; 7, *M. azadirachtae* DSM 23848^T^; 8, *M. lemovicicum* ViU22^T^; 9, *M. binotii* JCM 16365^T^.

Table S2. AAI and POCP values between two novel strains and their related type strains. AAI values were shaded in red and POCP values in yellow color. Strains: 1, *P. salitolerans* CJ85^T^; 2, *P. fandaimingii* HY82^T^; 3, *P. agarici* DSM 21798^T^; 4, *P. chengjingii* HY60^T^; 5, *P. humi* DSM 21799^T^; 6, *M. fluminis* CJ88^T^; 7, *M. azadirachtae* DSM 23848^T^; 8, *M. lemovicicum* ViU22^T^; 9, *M. binotii* JCM 16365^T^.

Table S3. Inhibition zone diameter (mm) of strains CJ85^T^ and CJ88^T^

Table S4. List of metabolic pathway modules based on KEGG database.

Fig. S1. Maximum-likelihood (A) and maximum-parsimony (B) phylogenetic trees of strain CJ85^T^ and related type strains based on 16S rRNA gene sequences. Bootstrap values greater than 70% are shown at branch points based on 1,000 replicated datasets. *Pseudoclavibacter soil* DSM 23366^T^ was used as an outgroup. Bar, 0.05 substitutions per nucleotide position for (A).

Fig. S2. Maximum-likelihood (A) and maximum-parsimony (B) phylogenetic trees of strain CJ88^T^ and related type strains based on 16S rRNA gene sequences. Bootstrap values greater than 70% are shown at branch points based on 1,000 replicated datasets. *Agrococcus jenensis* DSM 9580^T^ was used as an outgroup. Bar, 0.05 substitutions per nucleotide position for (A).

Fig. S3. Evaluation of the completeness of genome assembly results of strains CJ85^T^ and CJ88^T^. The completeness of the assembled genomes was evaluated with the datasets of *Microbacteriaceae* lineage.

Fig. S4. Phylogenomic tree of two novel strains and their related type strains based on 81 UBCGs. The phylogenomic tree was reconstructed using 158 type strains within the genera *Paramicrobacterium* and *Microbacterium*. Bootstrap value greater than 70% are shown at branch points based on maximum-likelihood analysis of 1,000 replicated datasets. The *Arthrobacter globiformis* NBRC 12137^T^ was used as an outgroup. Bar, 0.5 substitutions per nucleotide position

Fig. S5. Pangenome analysis of orthologous gene families within genera *Paramicrobacterium* and *Microbacterium*. The analysis depicts the distribution of orthologous gene families calculated separately for members of the genus *Paramicrobacterium*, members of the genus *Microbacterium*, and all strains from both genera combined. The numbers represent the counts of shared (core) or unique orthologous gene families among the strains. Strains: 1, *P. salitolerans* CJ85^T^; 2, *P. humi* DSM 21799^T^; 3, *P. chengjingii* HY60^T^; 4, *P. fandaimingii* HY82^T^; 5, *P. agarici* DSM 21798^T^; 6, *M. fluminis* CJ88^T^; 7, *M. binotii* JCM 16365^T^; 8, *M. lemovicicum* ViU22^T^; 9, *M. azadirachtae* DSM 23848^T^.

Fig. S6. Metabolic pathway module analysis of two novel strains and their related type strains based on KEGG annotation. The module descriptions were provided in Table S4.

Fig. S7. Comparative analysis of carbohydrate-active enzymes among two novel strains and their related type strains. (A) The bar plot showed the number of carbohydrate-active enzymes classified by CAZy enzyme classes. (B) The heatmap indicated the number of carbohydrate-active enzymes according to CAZy enzyme families.

Fig. S8. Comparative analysis of proteolytic enzymes among two novel strains and their related type strains.

Fig. S9. Transmission electron micrograph of strains CJ85^T^ (A) and CJ88^T^ (B). Cells were grown on TSA (A) and LB agar (B) for four days at 37°C. Bar, 1.0 μm

Fig. S10. Two-dimensional TLC of the total polar lipids from strain CJ85^T^ (A) and CJ88^T^ (B). Abbreviations: PG, phosphatidylglycerol; DPG, diphosphatidylglycerol; GL, glycolipid; L, polar lipid.
