# Supplementary material for: Paramicrobacterium salitolerans sp. nov. isolated from the agricultural soil and Microbacterium fluminis sp. nov. isolated from the Han River, South Korea
Source: J Microbiol. 2026 Mar 5;64(4):e2512014. doi: 10.71150/jm.2512014 (PMC13577007; doi:10.71150/jm.2512014)
Supplement: Supplementary file 3 [file jm-2512014-Supplementary-Fig-S2.pdf]

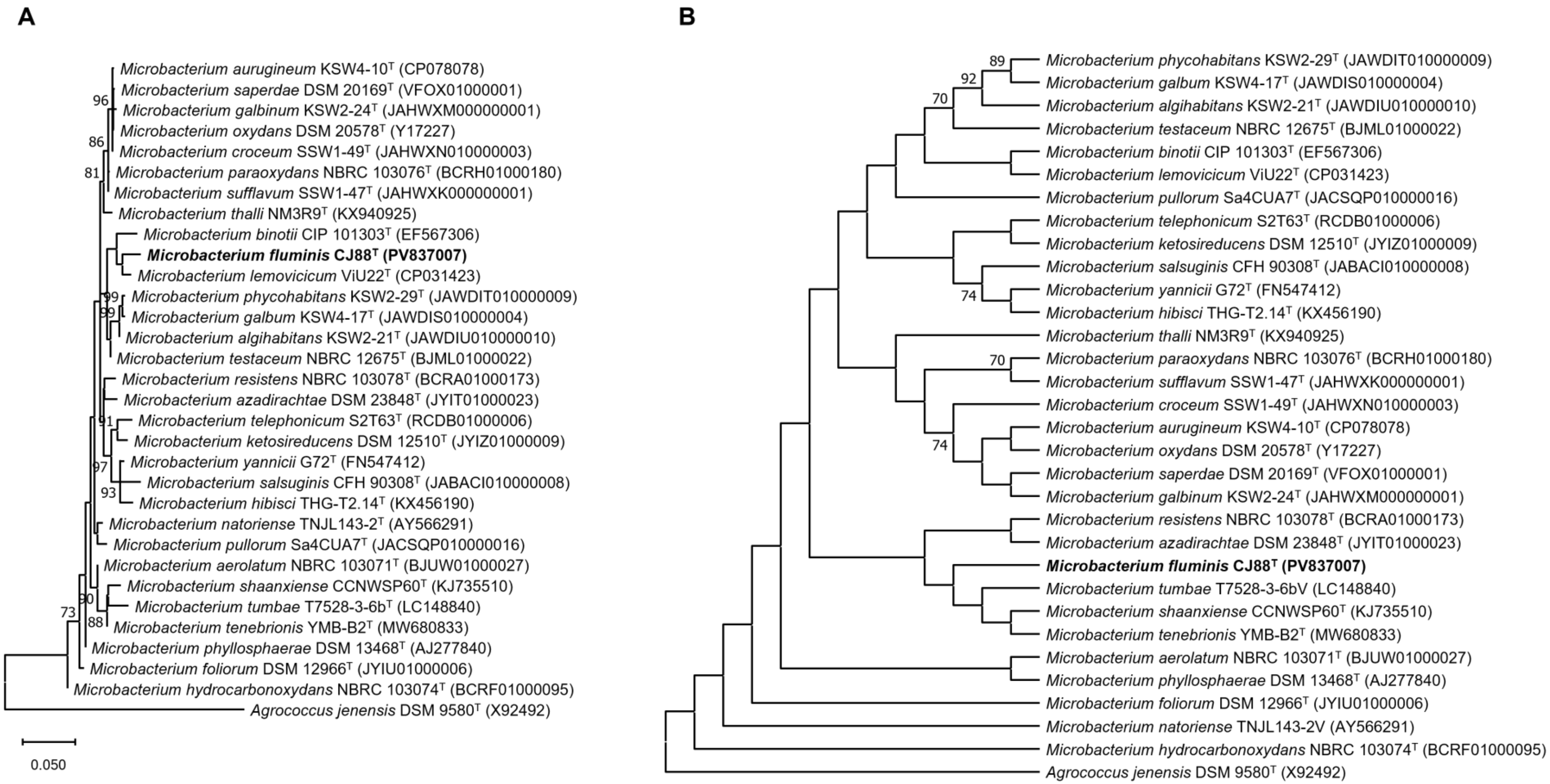

Fig. S2. Maximum-likelihood (A) and maximum-parsimony (B) phylogenetic trees of strain CJ88^T and related type strains based on 16S rRNA gene sequences. Bootstrap values greater than 70% are shown at branch points based on 1,000 replicated datasets. *Agrococcus jenensis* DSM 9580^T was used as an outgroup. Bar, 0.05 substitutions per nucleotide position for (A).
