# Supplementary material for: Paramicrobacterium salitolerans sp. nov. isolated from the agricultural soil and Microbacterium fluminis sp. nov. isolated from the Han River, South Korea
Source: J Microbiol. 2026 Mar 5;64(4):e2512014. doi: 10.71150/jm.2512014 (PMC13577007; doi:10.71150/jm.2512014)
Supplement: Supplementary file 4 [file jm-2512014-Supplementary-Fig-S3.pdf]

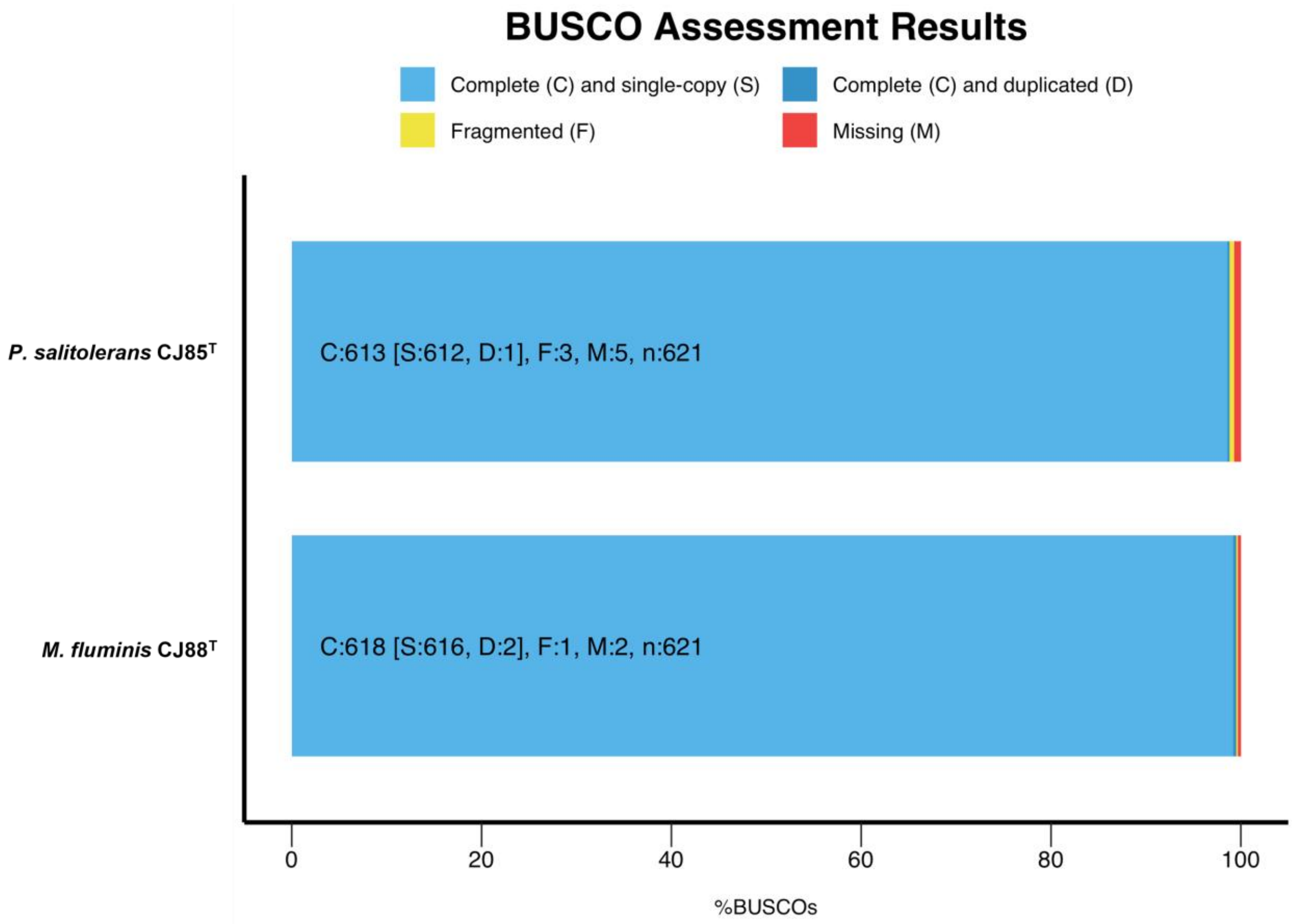

Fig. S3. Evaluation of the completeness of genome assembly results of strains CJ85^{T} and CJ88^{T}. The completeness of the assembled genomes was evaluated with the datasets of *Microbacteriaceae* lineage.
