# Supplementary material for: Paramicrobacterium salitolerans sp. nov. isolated from the agricultural soil and Microbacterium fluminis sp. nov. isolated from the Han River, South Korea
Source: J Microbiol. 2026 Mar 5;64(4):e2512014. doi: 10.71150/jm.2512014 (PMC13577007; doi:10.71150/jm.2512014)
Supplement: Supplementary file 5 [file jm-2512014-Supplementary-Fig-S4.pdf]

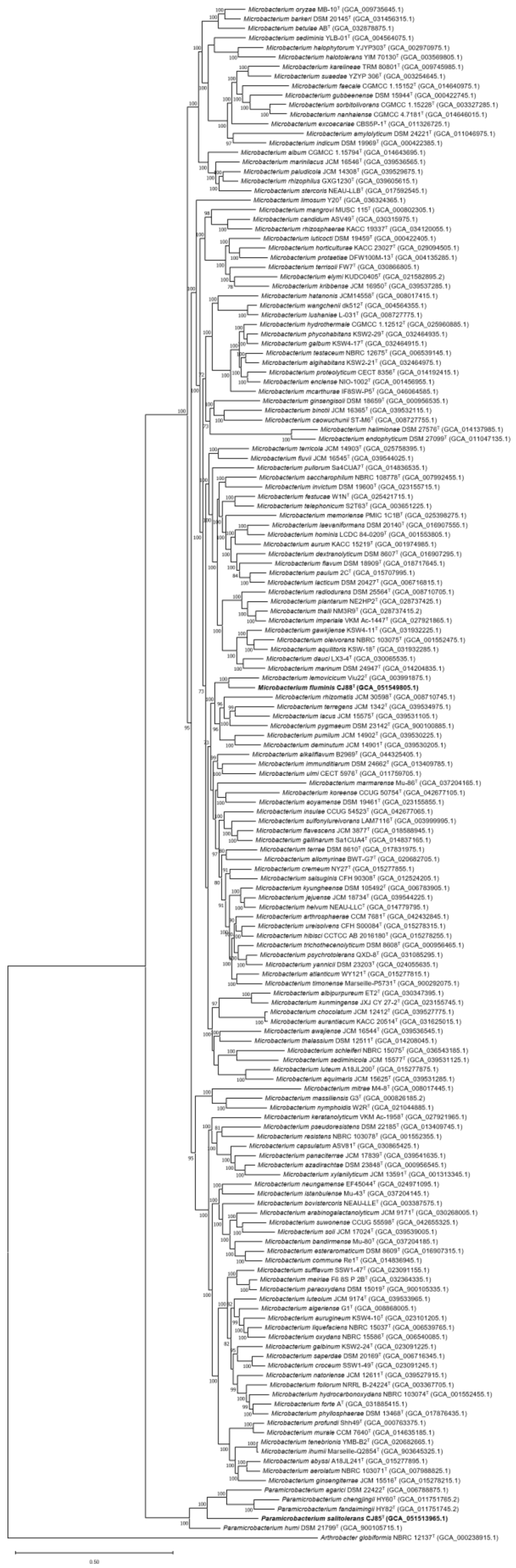

Fig. S4. Phylogenomic tree of two novel strains and their related type strains based on 81 UBCGs. The phylogenomic tree was reconstructed using 158 type strains within the genera *Paramicrobacterium* and *Microbacterium*. Bootstrap value greater than 70% are shown at branch points based on maximum-likelihood analysis of 1,000 replicated datasets. The *Arthrobacter globiformis* NBRC 12137^{T} was used as an outgroup. Bar, 0.5 substitutions per nucleotide position
