# Supplementary material for: Paramicrobacterium salitolerans sp. nov. isolated from the agricultural soil and Microbacterium fluminis sp. nov. isolated from the Han River, South Korea
Source: J Microbiol. 2026 Mar 5;64(4):e2512014. doi: 10.71150/jm.2512014 (PMC13577007; doi:10.71150/jm.2512014)
Supplement: Supplementary file 6 [file jm-2512014-Supplementary-Fig-S5.pdf]

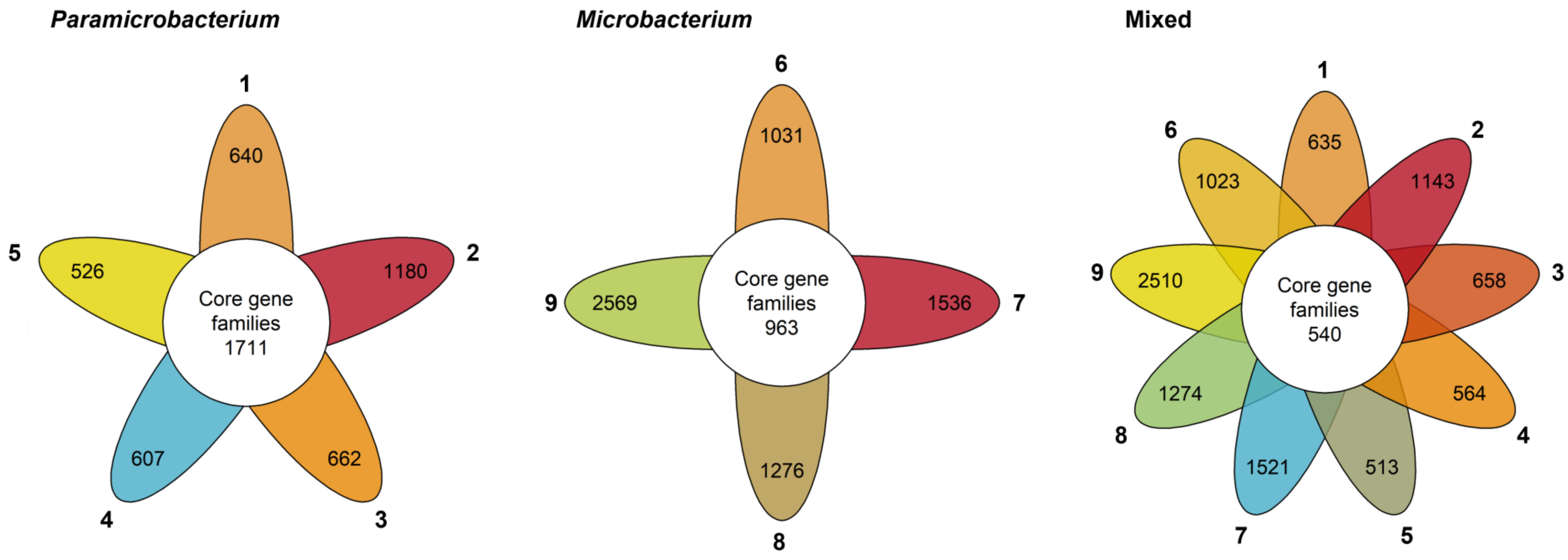

Fig. S5. Pangenome analysis of orthologous gene families within genera *Paramicrobacterium* and *Microbacterium*. The analysis depicts the distribution of orthologous gene families calculated separately for members of the genus *Paramicrobacterium*, members of the genus *Microbacterium*, and all strains from both genera combined. The numbers represent the counts of shared (core) or unique orthologous gene families among the strains.

Strains: 1, *P. salitolerans* CJ85^{T}; 2, *P. humi* DSM 21799^{T}; 3, *P. chengjingii* HY60^{T}; 4, *P. fandaimingii* HY82^{T}; 5, *P. agarici* DSM 21798^{T}; 6, *M. fluminis* CJ88^{T}; 7, *M. binotii* JCM 16365^{T}; 8, *M. lemovicicum* ViU22^{T}; 9, *M. azadirachtae* DSM 23848^{T}.
