# Supplementary material for: Paramicrobacterium salitolerans sp. nov. isolated from the agricultural soil and Microbacterium fluminis sp. nov. isolated from the Han River, South Korea
Source: J Microbiol. 2026 Mar 5;64(4):e2512014. doi: 10.71150/jm.2512014 (PMC13577007; doi:10.71150/jm.2512014)
Supplement: Supplementary file 7 [file jm-2512014-Supplementary-Fig-S6.pdf]

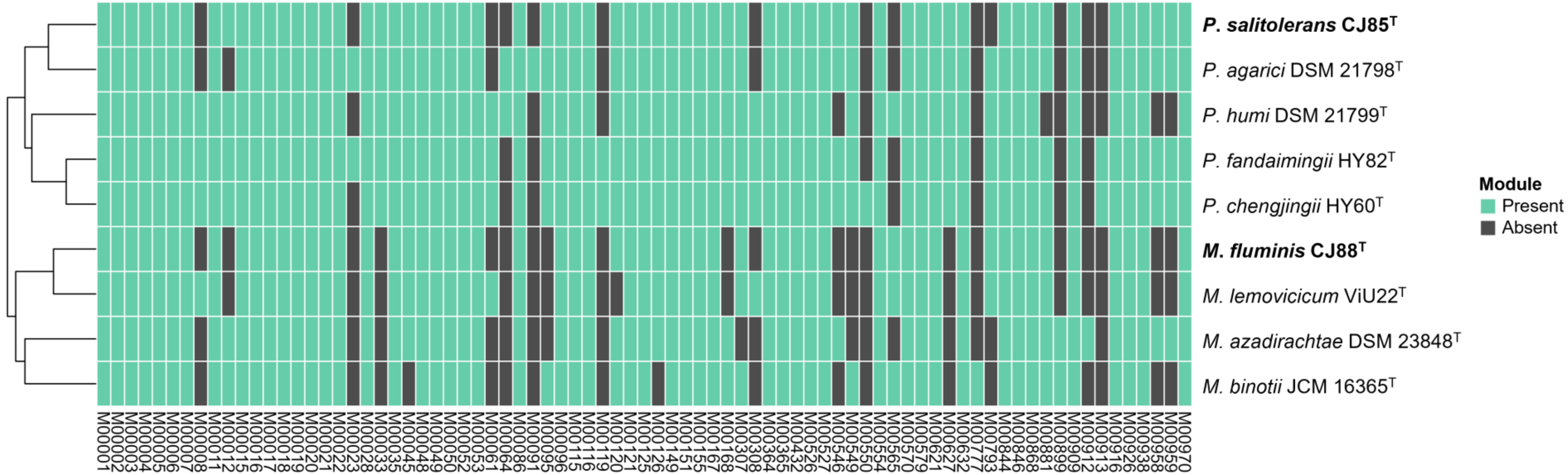

Fig. S6. Metabolic pathway module analysis of two novel strains and their related type strains based on KEGG annotation. The module descriptions were provided in Table S4.
