# Supplementary material for: Paramicrobacterium salitolerans sp. nov. isolated from the agricultural soil and Microbacterium fluminis sp. nov. isolated from the Han River, South Korea
Source: J Microbiol. 2026 Mar 5;64(4):e2512014. doi: 10.71150/jm.2512014 (PMC13577007; doi:10.71150/jm.2512014)
Supplement: Supplementary file 8 [file jm-2512014-Supplementary-Fig-S7.pdf]

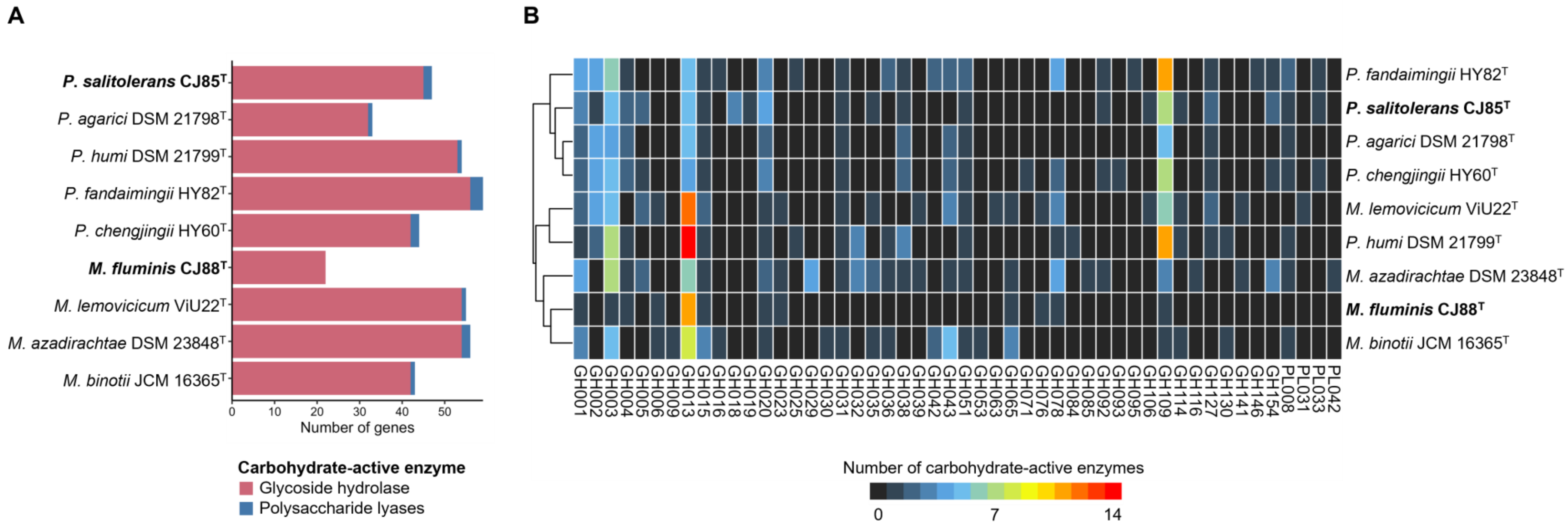

Fig. S7. Comparative analysis of carbohydrate-active enzymes among two novel strains and their related type strains. (A) The bar plot showed the number of carbohydrate-active enzymes classified by CAZy enzyme classes. (B) The heatmap indicated the number of carbohydrate-active enzymes according to CAZy enzyme families.
