# Supplementary material for: Paramicrobacterium salitolerans sp. nov. isolated from the agricultural soil and Microbacterium fluminis sp. nov. isolated from the Han River, South Korea
Source: J Microbiol. 2026 Mar 5;64(4):e2512014. doi: 10.71150/jm.2512014 (PMC13577007; doi:10.71150/jm.2512014)
Supplement: Supplementary file 10 [file jm-2512014-Supplementary-Fig-S9.pdf]

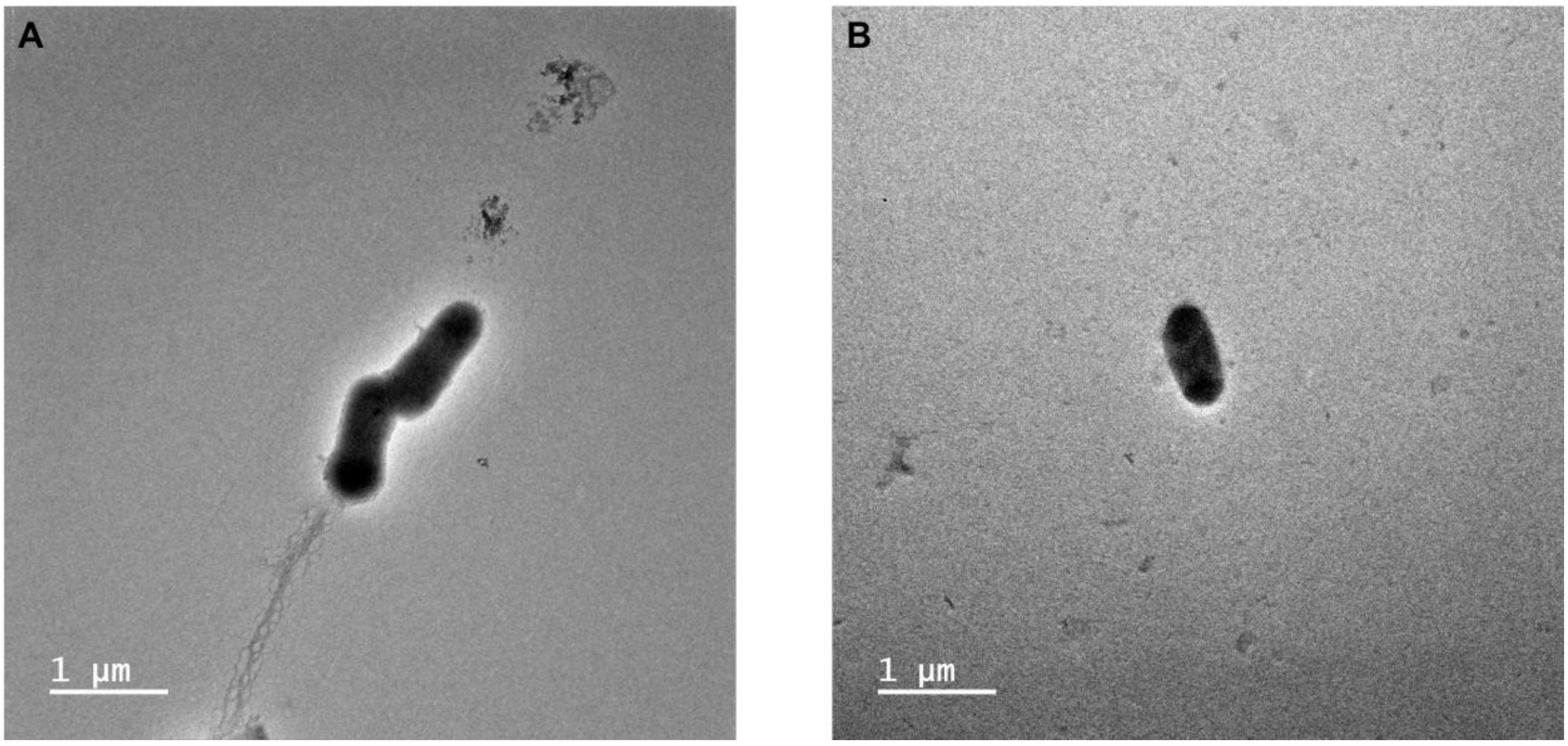

Fig. S9. Transmission electron micrograph of strains CJ85^T (A) and CJ88^T (B). Cells were grown on TSA (A) and LB agar (B) for four days at 37°C. Bar, 1.0 μm
