# Supplementary material for: Paramicrobacterium salitolerans sp. nov. isolated from the agricultural soil and Microbacterium fluminis sp. nov. isolated from the Han River, South Korea
Source: J Microbiol. 2026 Mar 5;64(4):e2512014. doi: 10.71150/jm.2512014 (PMC13577007; doi:10.71150/jm.2512014)
Supplement: Supplementary file 11 [file jm-2512014-Supplementary-Table-S1.pdf]

Table S1. ANI and dDDH values between two novel strains and their related type strains. ANI values were shaded in blue and dDDG values in green color.

Strains: 1, *P. salitolerans* CJ85^T; 2, *P. fandaimingii* HY82^T; 3, *P. agarici* DSM 21798^T; 4, *P. chengjingii* HY60^T; 5, *P. humi* DSM 21799^T; 6, *M. fluminis* CJ88^T; 7, *M. azadirachtae* DSM 23848^T; 8, *M. lemovicicum* ViU22^T; 9, *M. binotii* JCM 16365^T.

dDDH / ANI	1	2	3	4	5	6	7	8	9
**1**		19.8	20.6	19.2	20.3	19.6	19.3	19.1	19.1
**2**	76.8		22.4	28.8	19.7	19.9	19.6	19.4	19.1
**3**	77.8	80.1		21.8	20.2	19.1	19.5	19.1	19.6
**4**	76.3	84.8	79.6		19.1	19.9	19.9	18.8	19.6
**5**	76.9	76.1	76.8	75.4		19.5	19.5	19.4	18.8
**6**	71.1	70.3	71.0	69.8	72.6		20.3	23.1	20.8
**7**	70.8	69.9	70.6	69.7	72.0	75.9		20.2	19.9
**8**	71.0	70.3	71.0	69.6	72.3	80.4	75.3		20.1
**9**	70.9	70.1	70.5	69.6	72.0	76.9	74.7	76.1	
