# Supplementary material for: Paramicrobacterium salitolerans sp. nov. isolated from the agricultural soil and Microbacterium fluminis sp. nov. isolated from the Han River, South Korea
Source: J Microbiol. 2026 Mar 5;64(4):e2512014. doi: 10.71150/jm.2512014 (PMC13577007; doi:10.71150/jm.2512014)
Supplement: Supplementary file 12 [file jm-2512014-Supplementary-Table-S2.pdf]

Table S2. AAI and POCP values between two novel strains and their related type strains. AAI values were shaded in red and POCP values in yellow color.

Strains: 1, *P. salitolerans* CJ85^T; 2, *P. fandaimingii* HY82^T; 3, *P. agarici* DSM 21798^T; 4, *P. chengjingii* HY60^T; 5, *P. humi* DSM 21799^T; 6, *M. fluminis* CJ88^T; 7, *M. azadirachtae* DSM 23848^T; 8, *M. lemovicicum* ViU22^T; 9, *M. binotii* JCM 16365^T.

AAI \ POCP	1	2	3	4	5	6	7	8	9
**1**		74.6	76.5	75.4	68.3	51.2	50.7	50.2	50.9
**2**	78.6		78.6	79.6	68.9	49.5	48.8	50.9	49.4
**3**	79.7	84.1		76.4	72.2	51.3	49.2	50.2	49.9
**4**	78.5	89.0	84.1		65.9	48.7	47.4	49.0	47.5
**5**	76.1	75.7	76.3	75.8		52.5	49.6	51.7	50.2
**6**	63.3	62.9	63.2	62.9	63.7		58.8	69.9	64.2
**7**	62.9	62.3	62.7	62.3	63.3	69.3		57.0	54.7
**8**	63.1	62.8	62.9	62.5	63.5	78.7	68.2		63.6
**9**	62.8	62.7	62.8	62.4	63.6	72.7	68.3	71.4	
