# Supplementary material for: Paramicrobacterium salitolerans sp. nov. isolated from the agricultural soil and Microbacterium fluminis sp. nov. isolated from the Han River, South Korea
Source: J Microbiol. 2026 Mar 5;64(4):e2512014. doi: 10.71150/jm.2512014 (PMC13577007; doi:10.71150/jm.2512014)
Supplement: Supplementary file 13 [file jm-2512014-Supplementary-Table-S3.pdf]

Table S3. Inhibition zone diameter (mm) of strains CJ85^T and CJ88^T

Antibiotics	CJ85^T	Susceptible (S) / Intermediate (I) / Resistance (R)*	CJ88^T	Susceptible (S) / Intermediate (I) / Resistance (R)*
Chloramphenicol	35	S	26	S
Ciprofloxacin	32	S	6	R
Clindamycin	8	R	11	R
Erythromycin	6	R	52	S
Fosfomycin	13	I	6	R
Gentamicin	18	S	11	R
Linezolid	34	S	39	S
Rifampicin	6	R	8	R
Tetracycline	13	R	6	R
Trimethoprim	31	S	20	S
Vancomycin	17	S	22	S

*Susceptibility/Resistance results were recorded as susceptible (S), intermediate (I), or resistant (R) based on breakpoints for *Staphylococcus* spp. and *Enterococcus* spp. according to the CLSI zone diameter breakpoints table (35th edition).
