# Supplementary material for: Paramicrobacterium salitolerans sp. nov. isolated from the agricultural soil and Microbacterium fluminis sp. nov. isolated from the Han River, South Korea
Source: J Microbiol. 2026 Mar 5;64(4):e2512014. doi: 10.71150/jm.2512014 (PMC13577007; doi:10.71150/jm.2512014)
Supplement: Supplementary file 14 [file jm-2512014-Supplementary-Table-S4.pdf]

Table S4. List of metabolic pathway modules based on KEGG database.

Module	Description
M00001	Glycolysis (Embden-Meyerhof pathway), glucose => pyruvate
M00002	Glycolysis, core module involving three-carbon compounds
M00003	Gluconeogenesis, oxaloacetate => fructose-6P
M00004	Pentose phosphate pathway (Pentose phosphate cycle)
M00005	PRPP biosynthesis, ribose 5P => PRPP
M00006	Pentose phosphate pathway, oxidative phase, glucose 6P => ribulose 5P
M00007	Pentose phosphate pathway, non-oxidative phase, fructose 6P => ribose 5P
M00008	Entner-Doudoroff pathway, glucose-6P => glyceraldehyde-3P + pyruvate
M00011	Citrate cycle, second carbon oxidation, 2-oxoglutarate => oxaloacetate
M00012	Glyoxylate cycle
M00015	Proline biosynthesis, glutamate => proline
M00016	Lysine biosynthesis, succinyl-DAP pathway, aspartate => lysine
M00017	Methionine biosynthesis, aspartate => homoserine => methionine
M00018	Threonine biosynthesis, aspartate => homoserine => threonine
M00019	Valine/isoleucine biosynthesis, pyruvate => valine / 2-oxobutanoate => isoleucine
M00020	Serine biosynthesis, glycerate-3P => serine
M00021	Cysteine biosynthesis, serine => cysteine
M00022	Shikimate pathway, phosphoenolpyruvate + erythrose-4P => chorismate
M00023	Tryptophan biosynthesis, chorismate => tryptophan
M00028	Ornithine biosynthesis, glutamate => ornithine
M00033	Ectoine biosynthesis, aspartate => ectoine
M00035	Methionine degradation
M00045	Histidine degradation, histidine => N-formiminoglutamate => glutamate
M00048	De novo purine biosynthesis, PRPP + glutamine => IMP
M00049	Adenine ribonucleotide biosynthesis, IMP => ADP, ATP
M00050	Guanine ribonucleotide biosynthesis, IMP => GDP, GTP
M00052	Pyrimidine ribonucleotide biosynthesis, UMP => UDP/UTP, CDP/CTP
M00053	Deoxyribonucleotide biosynthesis, ADP/GDP/CDP/UDP => dATP/dGTP/dCTP/dUTP
M00061	D-Glucuronate degradation, D-glucuronate => pyruvate + D-glyceraldehyde 3P
M00064	ADP-L-glycero-D-manno-heptose biosynthesis
M00086	beta-Oxidation, acyl-CoA synthesis
M00091	Phosphatidylcholine (PC) biosynthesis, PE => PC
M00095	C5 isoprenoid biosynthesis, mevalonate pathway
M00096	C5 isoprenoid biosynthesis, non-mevalonate pathway
M00115	NAD biosynthesis, aspartate => quinolinate => NAD
M00116	Menaquinone biosynthesis, chorismate (+ polyprenyl-PP) => menaquinol
M00119	Pantothenate biosynthesis, valine/L-aspartate => pantothenate

M00120	Coenzyme A biosynthesis, pantothenate => CoA
M00121	Heme biosynthesis, plants and bacteria, glutamate => heme
M00125	Riboflavin biosynthesis, plants and bacteria, GTP => riboflavin/FMN/FAD
M00126	Tetrahydrofolate biosynthesis, GTP => THF
M00149	Succinate dehydrogenase, prokaryotes
M00151	Cytochrome bc1 complex respiratory unit
M00155	Cytochrome c oxidase, prokaryotes
M00167	Reductive pentose phosphate cycle, glyceraldehyde-3P => ribulose-5P
M00168	CAM (Crassulacean acid metabolism), dark
M00307	Pyruvate oxidation, pyruvate => acetyl-CoA
M00308	Semi-phosphorylative Entner-Doudoroff pathway, gluconate => glycerate-3P
M00364	C10-C20 isoprenoid biosynthesis, bacteria
M00365	C10-C20 isoprenoid biosynthesis, archaea
M00432	Leucine biosynthesis, 2-oxoisovalerate => 2-oxoisocaproate
M00526	Lysine biosynthesis, DAP dehydrogenase pathway, aspartate => lysine
M00527	Lysine biosynthesis, DAP aminotransferase pathway, aspartate => lysine
M00546	Purine degradation, xanthine => urea
M00549	Nucleotide sugar biosynthesis, glucose => UDP-glucose
M00550	Ascorbate degradation, ascorbate => D-xylulose-5P
M00554	Nucleotide sugar biosynthesis, galactose => UDP-galactose
M00565	Trehalose biosynthesis, D-glucose 1P => trehalose
M00570	Isoleucine biosynthesis, threonine => 2-oxobutanoate => isoleucine
M00579	Phosphate acetyltransferase-acetate kinase pathway, acetyl-CoA => acetate
M00621	Glycine cleavage system
M00627	β-Lactam resistance, Bla system
M00632	Galactose degradation, Leloir pathway, galactose => alpha-D-glucose-1P
M00793	dTDP-L-rhamnose biosynthesis
M00844	Arginine biosynthesis, ornithine => arginine
M00846	Siroheme biosynthesis, glutamyl-tRNA => siroheme
M00868	Heme biosynthesis, animals and fungi, glycine => heme
M00881	Lipoic acid biosynthesis, plants and bacteria, octanoyl-ACP => dihydrolipoyl-E2/H
M00909	UDP-N-acetyl-D-glucosamine biosynthesis, prokaryotes, glucose => UDP-GlcNAc
M00913	Pantothenate biosynthesis, 2-oxoisovalerate/spermine => pantothenate
M00916	Pyridoxal-P biosynthesis, R5P + glyceraldehyde-3P + glutamine => pyridoxal-P
M00926	Heme biosynthesis, bacteria, glutamyl-tRNA => coproporphyrin III => heme
M00938	Pyrimidine deoxyribonucleotide biosynthesis, UDP => dTTP
M00958	Adenine ribonucleotide degradation, AMP => Urate
M00959	Guanine ribonucleotide degradation, GMP => Urate
M00970	Proline degradation, proline => glutamate

M00777	Avermectin biosynthesis, 2-methylbutanoyl-CoA/isobutyryl-CoA => 6,8a-Seco-6,8a-deoxy-5-oxoavermectin 1a/1b aglycone => avermectin A1a/B1a/A1b/B1b
M00899	Thiamine salvage pathway, HMP/HET => TMP
M00912	NAD biosynthesis, tryptophan => quinolinate => NAD
